# Abnormal regional homogeneity as potential imaging biomarker for psychosis risk syndrome: a resting-state fMRI study and support vector machine analysis

**DOI:** 10.1038/srep27619

**Published:** 2016-06-08

**Authors:** Shuai Wang, Guodong Wang, Hailong Lv, Renrong Wu, Jingping Zhao, Wenbin Guo

**Affiliations:** 1 Mental Health Institute of the Second Xiangya Hospital, Central South University, The China National Clinical Research Center for Mental Health Disorders, National Technology Institute of Psychiatry, Key Laboratory of Psychiatry and Mental Health of Hunan Province, Changsha 410011, China; 2 Henan Key Laboratory of Biological Psychiatry, Henan Mental Hospital, The Second Affiliated Hospital of Xinxiang Medical University, Xinxiang 453002, China

## Abstract

Subjects with psychosis risk syndrome (PRS) have structural and functional abnormalities in several brain regions. However, regional functional synchronization of PRS has not been clarified. We recruited 34 PRS subjects and 37 healthy controls. Regional homogeneity (ReHo) of resting-state functional magnetic resonance scans was employed to analyze regional functional synchronization in these participants. Receiver operating characteristic curves and support vector machines were used to detect whether abnormal regional functional synchronization could be utilized to separate PRS subjects from healthy controls. We observed that PRS subjects showed significant ReHo decreases in the left inferior temporal gyrus and increases in the right inferior frontal gyrus and right putamen compared with the controls. No correlations between abnormal regional functional synchronization in these brain regions and clinical characteristics existed. A combination of the ReHo values in the three brain regions showed sensitivity, specificity, and accuracy of 88.24%, 91.89%, and 90.14%, respectively, for discriminating PRS subjects from healthy controls. We inferred that abnormal regional functional synchronization exists in the cerebrum of PRS subjects, and a combination of ReHo values in these abnormal regions could be applied as potential image biomarker to identify PRS subjects from healthy controls.

Psychosis risk syndrome (PRS) is the phase between the first noticeable changes in behavior and the appearance of overt psychotic symptoms^1^. High-risk subjects present basic (self-experienced deficits, stress tolerance disturbances, thought disorganization, and cognitive processing disorder)^2^, attenuated positive (suspiciousness, perceptual abnormalities, cognitive impairment, and magical thought content)^3^, and negative (simplistic thinking, decreased expression of emotion, odd appearance or thinking, and social isolation)^4^,^5^ symptoms, which are subtle and do not reach the psychosis thresholds. Individuals with PRS have approximately 30% to 40% risk of transiting to psychosis over the period of 12 months^6^. Thus, the probability of developing psychosis within the subsequent months is significantly greater for PRS subjects than for healthy population, and PRS has been considered a new diagnosis in the DSM-5 by several progressive studies^7^,^8^. However, these field advancements have been inconsistent. The validity of high risk criteria is still being discussed. The issue of false-positive results undermining the benefits of preventive interventions has been controversial. Thus, clinical need for reliable markers that could be used to help clinicians identify the subgroup of subjects that are specifically linked to the subsequent onset of psychosis is clear and urgent.

Neuroimaging techniques have been extensively employed to address the issue over the past decades^9^. The alterations in the structure^10^,^11^,^12^, function^13^,^14^,^15^, connectivity^16^, and neurochemistry^17^,^18^ of the cerebrum have been investigated in PRS subjects. The voxel-based structural neuroimaging studies on PRS subjects indicated that the hippocampal volume and gray matter were smaller in PRS subjects than in the controls^11^,^12^. Meanwhile, functional magnetic resonance imaging (fMRI) studies on PRS individuals showed significantly smaller differential activation between task-relevant and task-irrelevant stimuli in the frontal regions (anterior cingulate gyrus, interior frontal gyrus, and middle frontal gyrus) than the controls^14^. The PRS subjects also showed altered neural functions in the medial temporal cortex and the prefrontal regions during verbal encoding and correct recognition^15^.

Resting-state fMRI (rs-fMRI) has been drawing more attention as a new branch of this field in recent years. rs-fMRI can examine active regions in “resting-state” individuals, enabling the discovery of the core network responsible for internal modes of cognition^19^. Compared with task-related fMRI, rs-fMRI does not require attention to specific behaviorally relevant features of the external environment and is of interest for its potential to reveal the neural substrates of task-independent self-relevant information processing in schizophrenia and other psychoses^19^,^20^. In current rs-fMRI studies, a regional homogeneity (ReHo) method was applied to analyze the blood-oxygen-level-dependent (BOLD) signal in the brain and assumed that a given voxel was temporally similar to those of its neighbors. Kendall’s coefficient concordance (KCC) was used to measure the similarity or synchronization of the time series of a given voxel to those of its nearest neighbors in a voxel-wise way^21^, and thus, reflected a regional functional connectivity or synchronization and indicated the regional integration of information processing^22^,^23^. In fact, ReHo analysis has been successfully used to detect the abnormalities of regional functional synchronization in subjects with different psychiatric disorders, including ADHD^24^, depression^25^, and schizophrenia^26^,^27^. Compared with the controls, schizophrenic patients exhibited decreased ReHo in the precentral gyrus, middle occipital gyrus, and right parietal cortex, whereas increased ReHo in the medial prefrontal cortex and anterior insula^26^,^28^.

Previous studies reveal that PRS subjects exhibited abnormal brain structure in the hippocampus and left medial temporal cortex and abnormal function in the insula and medial temporal cortex^10^,^11^,^12^,^13^,^14^,^15^. However, whether PRS subjects exhibit abnormal regional functional synchronization during resting state remains unclear, which impedes the comprehensive understanding of the mechanisms of these abnormalities that contribute to the cognitive deficits and other psychotic symptoms in PRS subjects. Thus, we hypothesize that PRS subjects might exhibit abnormal regional functional synchronization that could be displayed by the ReHo analysis of the rs-fMRI. A case–control research was conducted between PRS subjects and healthy controls in the present study. Based on above-mentioned studies, we hypothesized that PRS subjects would exhibit abnormal regional functional synchronizations in certain brain regions, especially in the left inferior temporal gyrus, right inferior frontal gyrus, and right putamen. We also inferred that the abnormalities of regional functional synchronization in these brain regions might be considered as potential image biomarker for discriminating PRS subjects from healthy controls through support vector machine (SVM) analysis.

## Materials and Methods

### Subjects

We recruited 34 PRS subjects from the Second Xiangya Hospital of Central South University and 37 healthy controls unrelated to the PRS subjects from a community in Hunan Province of China. All participants were right-handed and group matched by age, gender, and years of education. PRS subjects were assessed, diagnosed using the Comprehensive Assessment for the At-Risk Mental State^3^ by two experienced clinicians, and excluded if they suffered from severe medical disorders, substance abuse, or had any contraindications for MRI. Controls were also excluded if they had any contraindications for MRI or their first-degree relatives suffered from psychotic disorders.

All participants were assessed with the Montgomery Asberg Depression Rating Scale (MADRS), Positive and Negative Syndrome Scale (PANSS), Trail Making Test (TMT), Brief Assessment of Cognition in Schizophrenia (BACS)^29^, Hopkins Verbal Learning Test-Revised (HVLT-R), and Brief Visuospatial Memory Test-Revised (BVMT-R) to measure and screen abnormal characteristics on recognition, affection, and behavior. In addition, PRS subjects were assessed with the Structured Interview for Psychosis-Risk Syndromes, Version 5.0 (SIPS) to evaluate symptom severity.

The study was conducted in accordance with the Helsinki Declaration^30^. All participants wrote their informed consents. The study was approved by the ethics committee of the Second Xiangya Hospital of Central South University.

### Scan acquisition

The rs-fMRI scanning was performed on a 3.0 T scanner (General Electric, Fairfield, Connecticut, USA). The participants were informed to lay supine in the scanner with their heads fixed with foam pads and a belt and remain motionless with eyes closed. Echo planar imaging (EPI) was used to acquire the resting-state functional images with the following parameters: repetition time/echo time (TR/TE) = 2000/30 ms, 33 axial slices, 64 × 64 matrix, 90° flip angle, 22 cm FOV, 4 mm section thickness, no slice gap, and 240 volumes.

### Data preprocessing

The statistical parametric mapping software (SPM8; http://fil.ion.ucl.ac.uk/spm/) and Data Processing Assistant for Resting-State fMRI (DPARSF)^31^ were employed for image preprocessing. The fMRI images were corrected for the acquisition delay between slices and head motion. All participants had less than 2 mm of translation in the *x*, *y*, or *z* and 2° of rotation in each axis. Then, the images were spatially normalized to the standard Montreal Neurological Institute (MNI) EPI template in SPM8 and resampled to 3 × 3 × 3 mm^3^. Finally, the images were linearly detrended and temporally band-pass-filtered (0.01 Hz to 0.08 Hz) to reduce the effect of low-frequency drifts and physiological high-frequency noise^32^,^33^.

### ReHo analysis

The REST software (http://resting-fmri.sourceforge.net) was used for the ReHo analysis. ReHo maps of each participant were obtained by calculating the KCC of the time series of a given voxel with those of its nearest neighbors (26 voxels)^21^. Then, the KCC among each voxel was divided to normalize the ReHo maps by the averaged KCC of the entire brain. The generated ReHo maps were spatially smoothed with a 4 mm full width at half maximum (FWHM) Gaussian kernel.

### Statistical analysis

The differences in age, sex, years of education, and clinical scales between the PRS subjects and the controls were estimated by SPSS 18.0 software using the Student’s *t* or Pearson’s chi-square (*χ*^2^) tests. Voxel-based comparisons of ReHo maps of the entire brain were performed using two-sample *t* tests. The resulting statistical maps were set at a threshold of *p* < 0.005 corrected for multiple comparisons using Gaussian random field (GRF) theory (min *z* > 2.807, cluster significance *p* < 0.005). The correlations between the ReHo values of these significant clusters and clinical scales in PRS subjects were performed by Pearson’s correlation analysis with a threshold of *p* < 0.05. The Bonferroni correction was applied to limit type I error in the multiple tests. Once the brain regions with significantly different ReHo values between the PRS subjects and the controls were observed, the receiver operating characteristic curve (ROC) analysis was used to detect whether these clusters could be utilized as markers to discriminate the PRS subjects from the controls at the group level.

### Classification analysis by using SVM

SVM using the LIBSVM^34^ software package (http://www.csie.ntu.edu.tw/~cjlin/libsvm/) ran in Matlab was applied to investigate the possibility of a combination of these clusters for discriminating the PRS subjects from the controls further. We included 34 PRS subjects and 37 controls in this analysis. Grid search method and Gaussian radial basis function kernels were used for parameter optimization, and a “leave-one-out” cross-validation approach was applied by the LIBSVM software to obtain the highest sensitivity and specificity.

## Results

### Participants and clinical baselines

Thirty-four PRS subjects and 37 healthy controls completed the resting-state fMRI scanning. However, three of the PRS subjects and five of the controls failed to finish the evaluation of clinical scales. The demographic data and clinical baselines of the participants are presented in Table 1. No differences in age, distribution of sex, years of education, and TMT scores were observed between PRS subjects and the controls (*p* > 0.05). However, MADRS and PANSS scores were significantly higher in PRS subjects (*p* < 0.05), whereas BACS, HVLT-R, and BVMT-R scores were significantly higher in the controls (*p* < 0.05).

### ReHo: group difference between PRS subjects and controls

**Figure 1:**
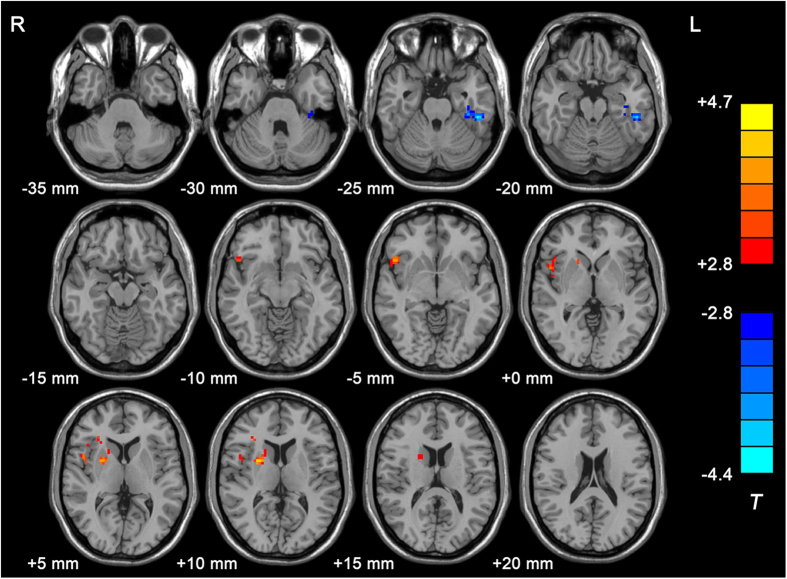
ReHo differences between PRS subjects and healthy controls. The color bar represents the *t* value of the group analysis of ReHo.

In the rs-fMRI images, PRS subjects showed significant ReHo decreases in the left inferior temporal gyrus (*t* = −4.256, corrected *p* < 0.005), but increases in the right inferior frontal gyrus (*t* = 4.011, corrected *p* < 0.005) and right putamen (*t* = 4.333, corrected *p* < 0.005) compared with the controls. The details are shown in Table 2 and Fig. 1.

### Correlations between ReHo values and clinical characteristics in PRS subjects

The ReHo values in the left inferior temporal gyrus were positively correlated with the disorganized symptoms of SIPS (*r* = 0.377, *p* = 0.037) and those in the right putamen were negatively correlated with the general symptoms of SIPS (*r* = − 0.431, *p* = 0.015). However, these correlations were no longer significant after the Bonferroni corrections (*p* < 0.05/5 = 0.01). No significant correlations were observed between the ReHo values in any brain region and age, years of education, and other clinical characteristics in PRS subjects (*p* > 0.05).

### Discriminating PRS subjects from controls

**Figure 2:**
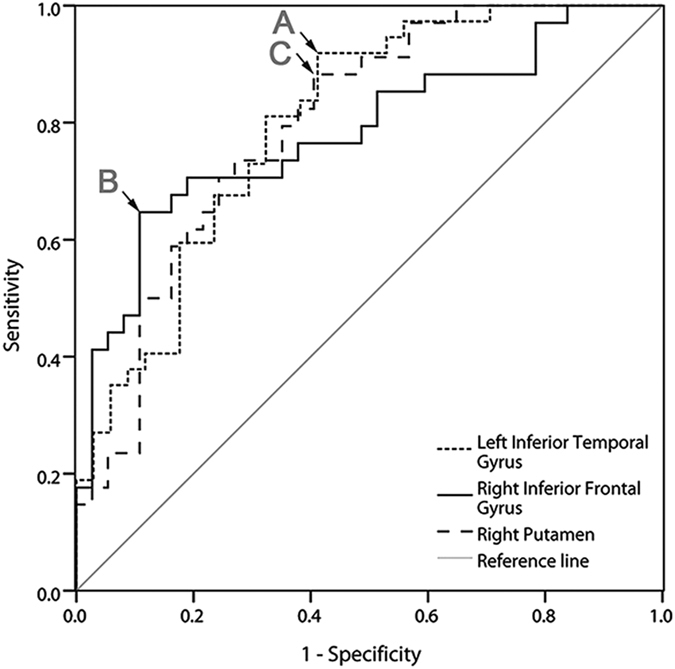
ROC of discriminating PRS subjects from healthy controls by using the ReHo values of the significantly different regions. Points (A–C) represent the cutoff value of ReHo in the left inferior temporal gyrus, right inferior frontal gyrus, and right putamen, respectively.

The left inferior temporal gyrus exhibited decreased ReHo values and the right inferior frontal gyrus and right putamen exhibited increased ReHo values in the PRS subjects. Therefore, we inferred that these brain regions might be utilized as markers to discriminate the PRS subjects from the controls. Then, the mean ReHo values in these three brain regions were extracted to verify this potential. ROC analyses showed that the area under the curve of the left inferior temporal gyrus was 0.800 with a cutoff point of −0.2022 and its diagnostic sensitivity and specificity were 91.89% and 58.82%, respectively, whereas the values were 0.783, 0.0138, 64.71%, and 89.19% for the right inferior frontal gyrus and 0.797, −0.1065, 88.24%, and 59.46% for right putamen, respectively (Table 3 and Fig. 2).

**Figure 3:**
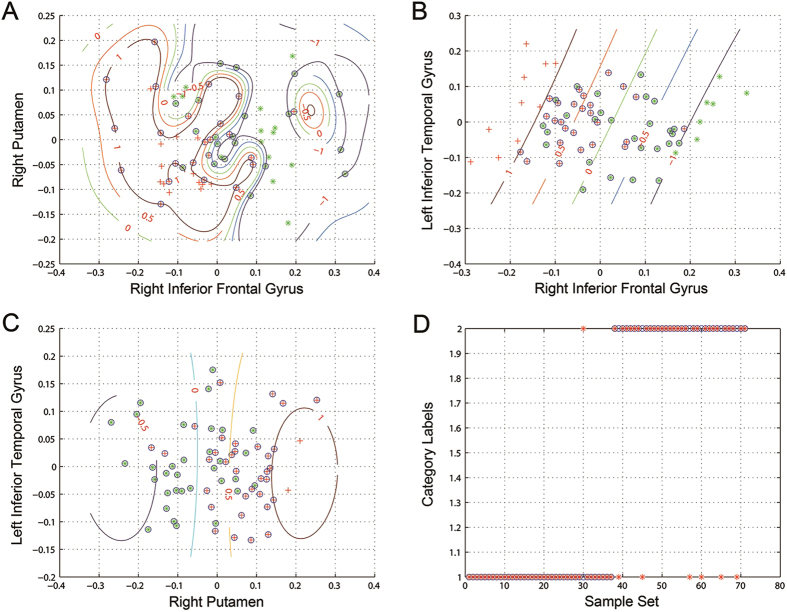
Visualization of classifications in SVM by using the combination of the ReHo values in the significantly different regions. (**A**) a combination of the right inferior frontal gyrus and right putamen; (**B**) a combination of the right inferior frontal gyrus and left inferior temporal gyrus; (**C**) a combination of the right putamen and left inferior temporal gyrus; (**D**) a combination of the three brain regions. In (**A–C**), red crosses represent the controls, green crosses represent the PRS, and blue circles represent the support vectors. In D, category 1 is the Control, category 2 is the PRS, red crosses represent the predicted set, and blue circles represent the actual set.

The SVM analyses were further conducted to determine whether the combination of the ReHo values in these brain regions could discriminate the PRS subjects from the controls with more optimal sensitivity and specificity. The combination of the right inferior frontal gyrus and right putamen showed a sensitivity of 82.35%, a specificity of 97.30%, and an accuracy of 90.14% (Fig. 3A). The combination of the right inferior frontal gyrus and left inferior temporal gyrus showed a sensitivity of 67.65%, a specificity of 89.19%, and an accuracy of 78.87% (Fig. 3B). The combination of the right putamen and left inferior temporal gyrus showed a sensitivity of 58.82%, a specificity of 91.89%, and an accuracy of 76.06% (Fig. 3C). Furthermore, the combination of the ReHo values in the three brain regions showed a sensitivity of 88.24%, a specificity of 91.89%, and an accuracy of 90.14% (Fig. 3D).

## Discussion

The key finding of this study is that PRS subjects exhibited a significantly lower ReHo in the left inferior temporal gyrus and significantly higher ReHo in the right inferior frontal gyrus and right putamen relative to the controls during the resting state. No correlations between the abnormal regional functional synchronization in these brain regions and the clinical characteristics existed. Further ROC analysis showed that the ReHo values in these brain regions, including the left inferior temporal gyrus, right inferior frontal gyrus, and right putamen, might not be used as potential markers to identify the PRS subjects from the controls, individually. However, the SVM analysis revealed that a combination of ReHo values in the three brain regions could serve as a right marker with a sensitivity of 88.24%, a specificity of 91.89%, and an accuracy of 90.14% for discriminating the PRS subjects from the controls.

The left inferior temporal gyrus is one of the most important brain regions involved in the pathophysiology of schizophrenia^35^ and has a crucial role in social cognition and emotional processes^36^. During the processes of verbal encoding and recognition, the PRS subjects exhibited altered brain function in the left temporal cortex^10^. Furthermore, reduced gray matter volume was observed in this region in PRS subjects compared with the controls or in the transition of psychosis from the PRS^37^,^38^,^39^. Moreover, patients with chronic and first-episode schizophrenia showed gray matter volume reductions in the left inferior temporal gyrus^40^,^41^,^42^. The structural and functional abnormalities in the left inferior temporal gyrus imply that the cognitive ability and emotional processes of the subjects with PRS has been injured. Our results were consistent with the evidence previously presented and revealed that PRS subjects exhibited a decreased regional functional synchronization in the left inferior temporal gyrus, which may represent a trait alteration of the population.

The inferior frontal gyrus is morphologically and functionally implicated to play a key role in the pathophysiology of schizophrenia^43^. The right inferior frontal gyrus in PRS subjects showed greater activity when performing the verbal fluency and N-back task^17^,^37^ and increases of regional functional synchronization in our study. Meanwhile, a reduced gray matter in the inferior frontal gyrus was also observed in the subjects with PRS^44^ and chronic schizophrenia^40^, who might show an established language dysfunction. However, the reduction of the gray matter density in the right inferior frontal gyrus was also observed in PRS subjects who later developed psychosis compared with those who did not^38^. Taken together with the findings of previous studies, we inferred that the expression of functional and structural abnormalities in the inferior frontal gyrus was not synchronous or proportional. In fact, the alteration of the regional functional synchronization in certain regions has been demonstrated to reflect physiological abnormalities associated with the early stage of schizophrenia, whereas changes of structure represent long-term and stable abnormalities in schizophrenic patients^45^,^46^.

When performing an analysis of group differences in the time versus frequency task contrast, patients with schizophrenia assumed relative hypoactivity in the putamen compared with the controls^47^. PRS subjects also showed a reduction of functional connectivity between the putamen and left thalamic and lenticular nuclei^48^. The critical involvement of the putamen is an integral part of the nigrostriatal system and highly reliant on dopaminergic neurotransmission^49^. In this regard, the concept of the putamen as a regulator of information to the thalamus is particularly pertinent to the pathophysiology of schizophrenia^50^. PRS subjects with a high probability of developing schizophrenia also exhibited wide-ranging neuropsychological deficits compared with those in patients with schizophrenia to a lesser degree. Our study revealed that the PRS subjects not only exhibited abnormal clinical symptoms, including sleep and movement disorders, which may be caused by the dysfunction of dopaminergic neurotransmission^51^,^52^, but also had an increased regional functional synchronization in the right putamen, implying abnormalities of regional integration of information processing in that region. However, whether the underlying mechanism of this increase is related to the abnormalities of neural neurotransmitter systems need further investigations.

Furthermore, the ROC results indicated that the sensitivity and specificity of these abnormal brain regions (individually) for determining the PRS subjects from the controls are not high, although the area under the curve of these regions was more than 0.7, which is an acceptable accuracy for established diagnostic indicators^53^. However, sensitivity or specificity less than 0.6 seems to be an indicator with poor accuracy^54^. Furthermore, subsequent SVM analyses show that the sensitivity, specificity, and accuracy were more than 0.8 in the combination of the right inferior frontal gyrus and right putamen and in the combination of these three brain regions for discriminating PRS subjects from healthy controls. Thus, we inferred that the combination of a decreased ReHo value in the left inferior temporal gyrus and an increased ReHo value in the right inferior frontal gyrus and right putamen could be employed as potential image biomarkers to identify the PRS subjects from the controls. However, no correlation was observed between the abnormal ReHo in any brain region and clinical scales of symptoms, indicating that the abnormal ReHo values in these regions could not serve as quantitative marker for the evaluation of clinical symptom severity.

Several limitations of the present study should be addressed. First, the structured clinical interviews were difficult and time-consuming and our sample size was inadequate and could easily have led to false-positive or false-negative results. Second, the differences of abnormal regional functional synchronization between the PRS subjects who will develop schizophrenia later and those who will not have not been clarified. Third, the use of the MNI template may be a potential confounder that will affect the accuracy of normalization for the Chinese subjects because the template was generated from a Caucasian population^45^. Finally, despite the limitations, the present study infers that abnormal regional functional synchronization exists in the cerebrum of PRS subjects, and a combination of ReHo values in these abnormal regions could be applied as potential image biomarker to identify PRS subjects from healthy controls.

## Additional Information

**How to cite this article**: Wang, S. *et al.* Abnormal regional homogeneity as potential imaging biomarker for psychosis risk syndrome: a resting-state fMRI study and support vector machine analysis. *Sci. Rep.* **6**, 27619; doi: 10.1038/srep27619 (2016).

## Figures and Tables

**Table 1:** Demographic data and clinical baselines of the participants.

Characteristics	PRS	Controls	*t*/*χ*^2^	*p*
Sample size	34	37		
Gender (male/female)	21/13	18/19	1.231	0.341
Age (years)	21.50 ± 3.53	20.76 ± 3.08	0.948	0.346
Years of education (years)	12.50 ± 3.82	13.89 ± 2.00	−1.945	0.056
MADRS	13.29 ± 6.50	0.85 ± 1.73	10.331	<0.001
PANSS	64.87 ± 10.96	30.79 ± 1.36	16.912	<0.001
Positive	12.38±4.06	6.53 ± 2.04	7.363	<0.001
Negative	15.16 ± 6.56	6.47 ± 1.99	7.199	<0.001
General	33.28 ± 10.68	15.22 ± 4.76	8.817	<0.001
TMT	41.79 ± 18.66	36.92 ± 11.22	1.307	0.196
BACS	53.96 ± 9.49	60.97 ± 10.72	−2.742	0.008
HVLT-R	22.79 ± 6.44	27.05 ± 3.87	−3.110	0.003
BVMT-R	20.41 ± 11.63	27.65 ± 5.81	−3.272	0.002
SIPS	35.13 ± 11.23			
Positive	9.52 ± 3.14			
Negative	13.10 ± 6.72			
Disorganized	6.23 ± 3.23			
General	6.29 ± 3.43			

PRS, Psychosis Risk Syndrome; MADRS, Montgomery Asberg Depression Rating Scale; PANSS, Positive and Negative Syndrome Scale; TMT, Trail Making Test; BACS, Brief Assessment of Cognition in Schizophrenia; HVLT-R, Hopkins Verbal Learning Test-Revised; BVMT-R, Brief Visuospatial Memory Test-Revised; SIPS, Structured Interview for Psychosis-Risk Syndromes.

**Table 2:** Brain regions with significantly different ReHo values between the PRS subjects and the healthy controls.

Brain region	ReHo values		Number of voxels	*t*	*p*	Peak MNI coordinate		
	PRS	Controls				X	Y	Z
Deceased (PRS < Controls)								
Left Inferior Temporal Gyrus	−0.197 ± 0.086	−0.092 ± 0.084	51	−4.256	<0.005	−48	−33	−24
Increased (PRS > Controls)								
Right Inferior Frontal Gyrus	0.050 ± 0.119	−0.072 ± 0.100	64	4.011	<0.005	48	18	−6
Right Putamen	−0.026 ± 0.080	−0.117 ± 0.078	66	4.333	<0.005	24	3	9

PRS, Psychosis Risk Syndrome; ReHo, Regional Homogeneity; MNI, Montreal Neurological Institute.

**Table 3:** Discriminating the PRS subjects from the healthy controls by ROC analyses. AUC, Area Under the Curve.

Brain region	AUC	Cut-off value	Sensitivity	Specificity
Left Inferior Temporal Gyrus	0.800	−0.2022	91.89%	58.82%
Right Inferior Frontal Gyrus	0.783	0.0138	64.71%	89.19%
Right Putamen	0.797	−0.1065	88.24%	59.46%
